# Impact of Sulfated Hyaluronan on Bone Metabolism in Diabetic Charcot Neuroarthropathy and Degenerative Arthritis

**DOI:** 10.3390/ijms232315146

**Published:** 2022-12-02

**Authors:** Sabine Schulze, Christin Neuber, Stephanie Möller, Ute Hempel, Lorenz C. Hofbauer, Klaus-Dieter Schaser, Jens Pietzsch, Stefan Rammelt

**Affiliations:** 1 University Center for Orthopaedics, Trauma and Plastic Surgery, University Hospital Carl Gustav Carus, Technische Universität Dresden, 01307 Dresden, Germany; 2 Center for Translational Bone, Joint and Soft Tissue Research, Medical Faculty, Technische Universität Dresden, 01307 Dresden, Germany; 3 Helmholtz-Zentrum Dresden-Rossendorf, Institute of Radiopharmaceutical Cancer Research, Department of Radiopharmaceutical and Chemical Biology, 01328 Dresden, Germany; 4 Biomaterials Department, INNOVENT e. V., Prüssingstrasse 27B, 07745 Jena, Germany; 5 Institute of Physiological Chemistry, Medical Faculty, Technische Universität Dresden, 01307 Dresden, Germany; 6 Department of Medicine III, University Hospital Carl Gustav Carus, Technische Universität Dresden, 01307 Dresden, Germany; 7 Center for Healthy Aging, University Hospital Carl Gustav Carus, Technische Universität Dresden, 01307 Dresden, Germany; 8 Faculty of Chemistry and Food Chemistry, School of Science, Technische Universität Dresden, 01069 Dresden, Germany

**Keywords:** Charcot neuroarthropathy, diabetes mellitus, ankle arthritis, osteoblasts, osteoclasts, sulfated hyaluronan

## Abstract

Bone in diabetes mellitus is characterized by an altered microarchitecture caused by abnormal metabolism of bone cells. Together with diabetic neuropathy, this is associated with serious complications including impaired bone healing culminating in complicated fractures and dislocations, especially in the lower extremities, so-called Charcot neuroarthropathy (CN). The underlying mechanisms are not yet fully understood, and treatment of CN is challenging. Several in vitro and in vivo investigations have suggested positive effects on bone regeneration by modifying biomaterials with sulfated glycosaminoglycans (sGAG). Recent findings described a beneficial effect of sGAG for bone healing in diabetic animal models compared to healthy animals. We therefore aimed at studying the effects of low- and high-sulfated hyaluronan derivatives on osteoclast markers as well as gene expression patterns of osteoclasts and osteoblasts from patients with diabetic CN compared to non-diabetic patients with arthritis at the foot and ankle. Exposure to sulfated hyaluronan (sHA) derivatives reduced the exaggerated calcium phosphate resorption as well as the expression of genes associated with bone resorption in both groups, but more pronounced in patients with CN. Moreover, sHA derivatives reduced the release of pro-inflammatory cytokines in osteoclasts of patients with CN. The effects of sHA on osteoblasts differed only marginally between patients with CN and non-diabetic patients with arthritis. These results suggest balancing effects of sHA on osteoclastic bone resorption parameters in diabetes.

## 1. Introduction

The pathologic changes to bone in diabetes mellitus are multifactorial and not yet completely understood. In Charcot neuroarthropathy (CN), the physiological bone architecture is disrupted, culminating in pathologic fractures, especially at the foot and ankle [1]. Due to peripheral neuropathy with impairment in pain perception, these fractures often remain unnoticed by patients, leading to deformities, ulceration, infection and amputation [2,3,4]. The increased inflammatory state in diabetes is highly associated with altered bone architecture and neuropathy [5] influencing bone stiffness and strength [6] as also known from inflammation-induced bone loss in cancer patients [7,8]. However, it is still uncertain whether local microtrauma and dysregulated bone resorption cause the inflammation or whether the enhanced inflammatory state leads to structural fragility and an increased fracture risk in CN [9].

The physiological stability of bone derives from the inorganic mineral calcium phosphate phase, mainly hydroxyapatite and its precursors, which is fundamental for load-bearing. Flexibility and elasticity are provided by the organic extracellular matrix (ECM), which is composed of collagen, non-collagenous matrix proteins and glycosaminoglycans (GAG).

The ECM is a cell-derived, non-cellular network embedding cells in a cluster. It orchestrates the interaction of cells with each other and with growth factors, inducing several signal transduction pathways that regulate cell proliferation and differentiation [10]. Because of their negative charges, GAG can bind growth factors, cytokines and chemokines resulting in accumulation of these molecules and simultaneous shielding from degradation [11]. Thus, depending on the binding site, GAG either prolong and amplify or inhibit the signal transduction cascades. These properties make GAG potential candidates for the treatment of pathological conditions and stimulation of physiological responses in impaired tissues [8].

GAG-based artificial extracellular matrices (aECM) with sulfated hyaluronan (sHA) as the key component promote osteogenic differentiation of human mesenchymal stromal cells (hMSC) in vitro [12] by altering the assembly of components of the natural ECM [13]. Consequently, the activity of the osteogenic marker tissue non-specific alkaline phosphatase (TNAP) increases and osteoblasts enhance their calcium phosphate deposition. In vivo experiments revealed increased bone volume in critical-size femur defects in healthy rats [14] as well as in diabetic rats through sHA derivatives [15]. In addition to its stimulating effect on osteoblasts, sHA inhibits the formation of multinucleated cells in a murine osteoclast cell line [16]. However, there is insufficient knowledge about these mechanisms in humans.

This patient-based study addresses the impact of sHA on the osteoclastogenesis of peripheral blood mononuclear cells (PBMC) and osteogenic markers of osteoblasts isolated from bone fragments. Blood and bone fragments were collected from patients who underwent reconstructive foot and ankle surgery for either arthritis or diabetic CN. Furthermore, the effect of different degrees of sulfation of sHA derivatives on specific markers for osteoblasts and osteoclasts and on gene expression was determined in order to identify biomaterials with osteogenic potential in impaired bone metabolism through diabetes.

## 2. Results

### 2.1. Osteoclasts

**Figure 1:**
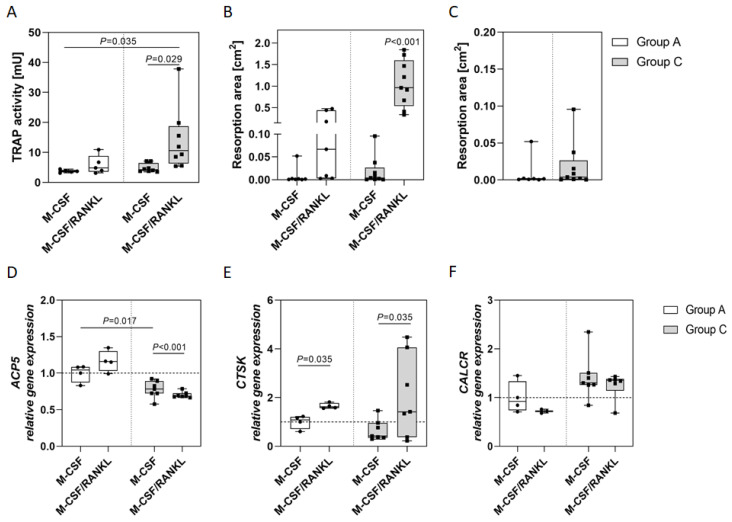
TRAP and resorption activity and gene expression of PBMC derived either from arthritis (group A) or Charcot patients (group C), respectively. (**A**) TRAP activity was determined in PBMC either treated with M-CSF only or with M-CSF and RANKL after 14 days of culture (group A: n = 5; group C: n = 8). (**B**) Resorption activity was performed by culturing PBMC on a cell-derived calcium phosphate matrix. After 14 days of culture, calcium phosphate was stained black by von Kossa silver staining. Wells were scanned and black (calcium phosphate) and white (resorbed) areas were quantified (group A: n = 7; group C: n = 9). (**C**) Comparison of initial resorption activity of both patient groups after M-CSF only treatment showing higher initial resorption activity without RANKL in group C than in group A (*p* = 0.48). *ACP5* (**D**), *CTSK* (**E**) and *CALCR* (**F**) expression were determined by qPCR after 14 days of culture (group A: n = 4; group C: n = 7). Results are shown relative to *GAPDH* expression and were normalized to group A M-CSF and were calculated using the CT method. All data are provided as median with interquartile range and min/max values; statistical analyses for (**A**,**B**,**D**–**F**) were performed using ANOVA and Tukey post-hoc test; statistical analysis for (**C**) was performed using *t* test.

Treatment of PBMC with macrophage colony-stimulating factor (M-CSF) alone (unstimulated control) showed similar initial values of tartrate-resistant acid phosphatase (TRAP) activity in both arthritis (group A) and Charcot patients (group C), with slightly elevated values in the latter (range from 3.166 to 4.477 for group A (median 3.767 mU) and from 3.531 to 7.136 for group C (median 4.829 mU); Figure 1A). Receptor activator of nuclear factor κ ligand (RANKL) treatment resulted in increased TRAP activity in both groups that was significant in group C. In addition, the resorption activity significantly increased by a factor of ten in group C after the administration of RANKL (Figure 1B). Direct comparison of the resorption in presence of M-CSF only indicates a higher initial resorption activity for PBMC in group C (Figure 1C).

The expression of osteoclast-specific genes *ACP5*, *CTSK* and *CALCR* was determined for both groups. The initial *ACP5* expression was significantly increased in group A compared to group C and was enhanced by RANKL treatment in group A (Figure 1D). RANKL significantly reduced the *ACP5* expression in group C. The initial *CTSK* expression in group C was similar compared to group A, but was significantly enhanced by the addition of RANKL in both patient groups (Figure 1E). The initial *CALCR* expression was insignificantly higher in group C (Figure 1F). RANKL had no significant effect on the expression of *CALCR* in both patient groups.

**Figure 2:**
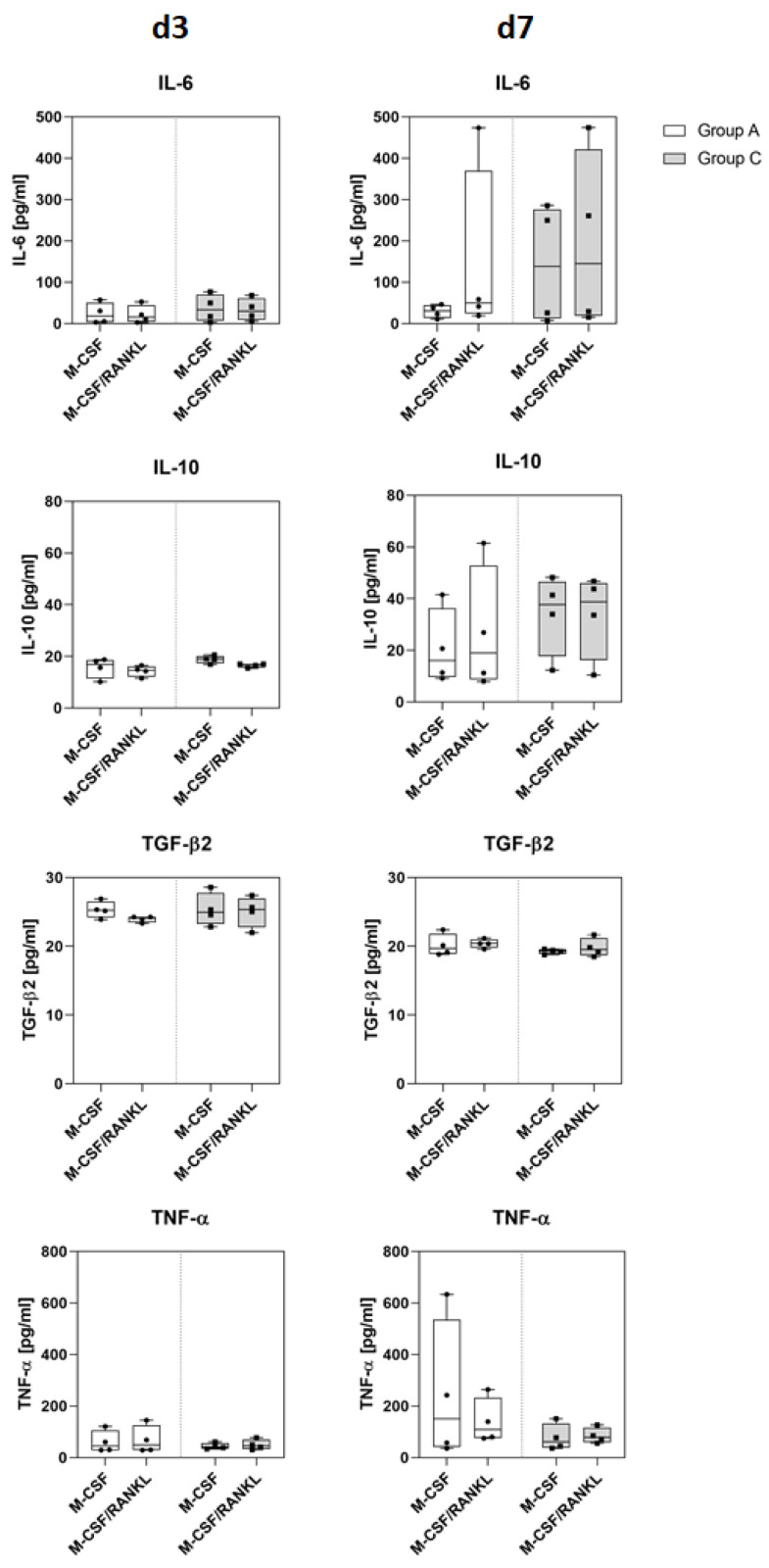
Secretion of inflammatory cytokines in PBMC of group A and group C. Cell culture supernatants were collected on day 3 and 7 from cells treated with either M-CSF or M-CSF/RANKL (n = 4). IL-6, IL-10, TGF-β2 and TNF-α were quantified by ELISA. Data are given as median with interquartile range and min/max values, statistical analyses were performed using ANOVA and Tukey post-hoc test.

The release of pro- and anti-inflammatory cytokines in PBMC is depicted in Figure 2. On day 3 of cell culture, group C PBMC secreted higher concentrations of IL-6 than group A PBMC independent from M-CSF or M-CSF/RANKL treatment. On day 7, the IL-6 concentration was enhanced in both groups after M-CSF/RANKL treatment. Although there was a clear trend towards higher IL-6 secretion in group C following M-CSF-treatment, with the numbers available the differences were not statistically significant. IL-10 was increased in group C under both conditions, M-CSF and M-CSF/RANKL, on day 3 and doubled until day 7. The IL-10 secretion in group A increased patient-specifically from day 3 to day 7. Both groups showed similar TGF-β2 concentrations on day 3, which decreased on day 7, independent of treatment. By trend, the TNF-α secretion was four to five fold lower in group C in comparison to group A at both time points. However, the cytokine concentrations did not differ significantly between both groups due to high interindividual variability.

### 2.2. Osteoblast Gene Expression

**Figure 3:**
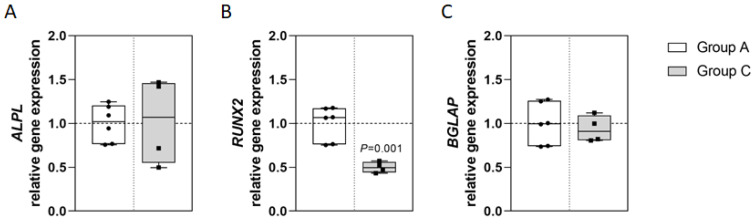
Gene expression in osteoblasts. Osteoblasts of both patient groups were cultured for 14 days (group A: n = 6; group C: n = 4) before RNA isolation and reverse transcription. Quantitative real-time PCR revealed the gene expressions of *ALPL* (**A**), *RUNX2* (**B**) and *BGLAP* (**C**). Gene expression was normalized to *GAPDH* expression and analyzed using the CT method. Data are provided relative to group A control and as median with interquartile range and min/max values; statistical analyses were performed using ANOVA and Tukey post-hoc test.

The TNAP encoding gene *ALPL* showed similar expression rates in both groups (Figure 3A). *RUNX2*, encoding Runx2, a master transcription factor for osteogenic lineage commitment of mesenchymal osteoblast precursor cells, was expressed significantly less in group C (Figure 3B). The osteocalcin encoding gene *BGLAP* was expressed at similar levels in both groups (Figure 3C).

### 2.3. Effect of Sulfated Hyaluronan Derivatives on Osteoclasts and Osteoblasts

**Figure 4:**
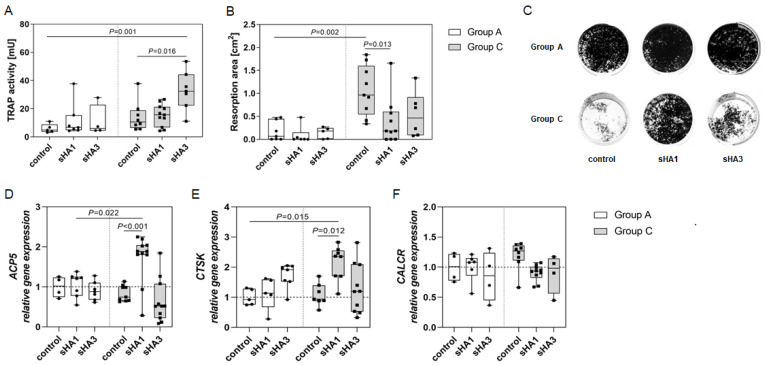
Effect of sHA on TRAP activity, calcium phosphate resorption and gene expression in osteoclasts. PBMC were cultured on a calcium phosphate matrix. (**A**–**C**) After 14 days of culture with M-CSF/RANKL and sHA (200 µg/mL) TRAP activity (**A**) and resorption activity (**B**) were analyzed. (**C**) Representative images of resorption assays in 24-well plates of M-CSF/RANKL-treated PBMC of an arthritis (upper row) and Charcot patient (lower row) after 14 days of culture. Strong resorption of Charcot patients was diminished after sHA administration. Black: silver-stained calcium phosphate; white: resorbed areas. (**D**–**F**) After 14 days of culture cells were lysed for RNA isolation followed by reverse transcription and quantitative real-time PCR to evaluate the expression of *ACP5* (**D**), *CTSK* (**E**) and *CALCR* (**F**). Gene expression was normalized to *GAPDH* expression and calculated using the CT method. Data are provided as median with interquartile range and min/max values, statistical analyses were performed using ANOVA and Tukey post-hoc test.

Treatment with sHA increased TRAP activity in PBMC of both patient groups that were cultured with M-CSF/RANKL (Figure 4A). Incubation with sHA3 induced a doubling of TRAP activity in group C, contrary to group A, compared to the untreated control. Calcium phosphate resorption dropped in group A after sHA administration, yet not significantly (Figure 4B). Group C showed significantly increased resorption activity compared to group A and the addition of sHA almost halved the resorption activity of the cells from CN patients (Figure 4B,C). Figure 4C shows representative von Kossa staining of PBMC after 14 days culture on a calcium phosphate matrix indicating increased bone resorption in group C that reduced substantially in the presence of sHA1.

PBMC, differentiated with M-CSF/RANKL and treated with sHA for 14 days, were analyzed for gene expression. sHA1 induced a significant increase of *ACP5* expression in group C only (Figure 4D). *CTSK* expression was enhanced by both sHA derivatives in both patient groups. This increase was significant for sHA1 treatment in group C (Figure 4E). *CALCR* expression was higher in the controls of group C and decreased after sHA treatment (Figure 4F).

**Figure 5:**
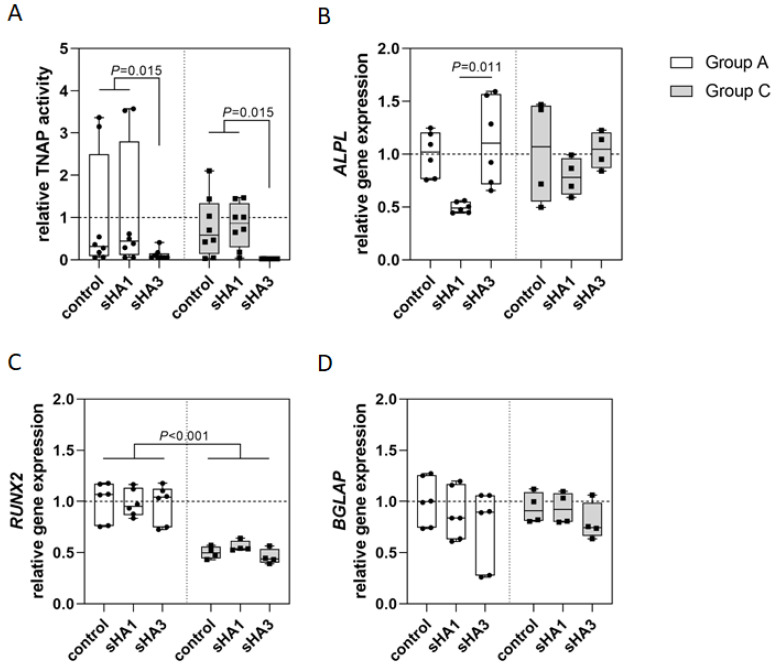
Impact of sHA on osteoblasts after 14 days of sHA treatment. (**A**) TNAP activity was quantified in cells of eight patients per group. The untreated control of group A was set to 1. TNAP activity was reduced significantly after sHA3 treatment. Expression of the osteogenic marker genes *ALPL* (**B**), *RUNX2* (**C**) and *BGLAP* (**D**) was evaluated by quantitative real-time PCR. Gene expression of the target genes was normalized to *GAPDH* expression and calculated using the CT method. Data are shown as median with interquartile range and min/max values, statistical analyses were performed using ANOVA and Tukey post-hoc test.

Osteoblasts isolated from both patient groups showed similar values of TNAP activity in the untreated control and when sHA1 was applied (Figure 5A). sHA3 administration resulted in significantly reduced TNAP activity in both groups (Figure 5A). *ALPL* expression was diminished by sHA1 but not sHA3 in both groups (Figure 5B). *RUNX2* expression was significantly decreased in group C compared to group A (Figure 5C) but was not affected by sHA treatment in both patient groups (Figure 5C). *BGLAP* expression was only decreased by sHA treatment in both groups by trend (Figure 5D), with a higher effect in osteoblasts treated with sHA3 compared to sHA1. Taken together, sHA only had marginal effects on gene expression of osteoblasts.

**Figure 6:**
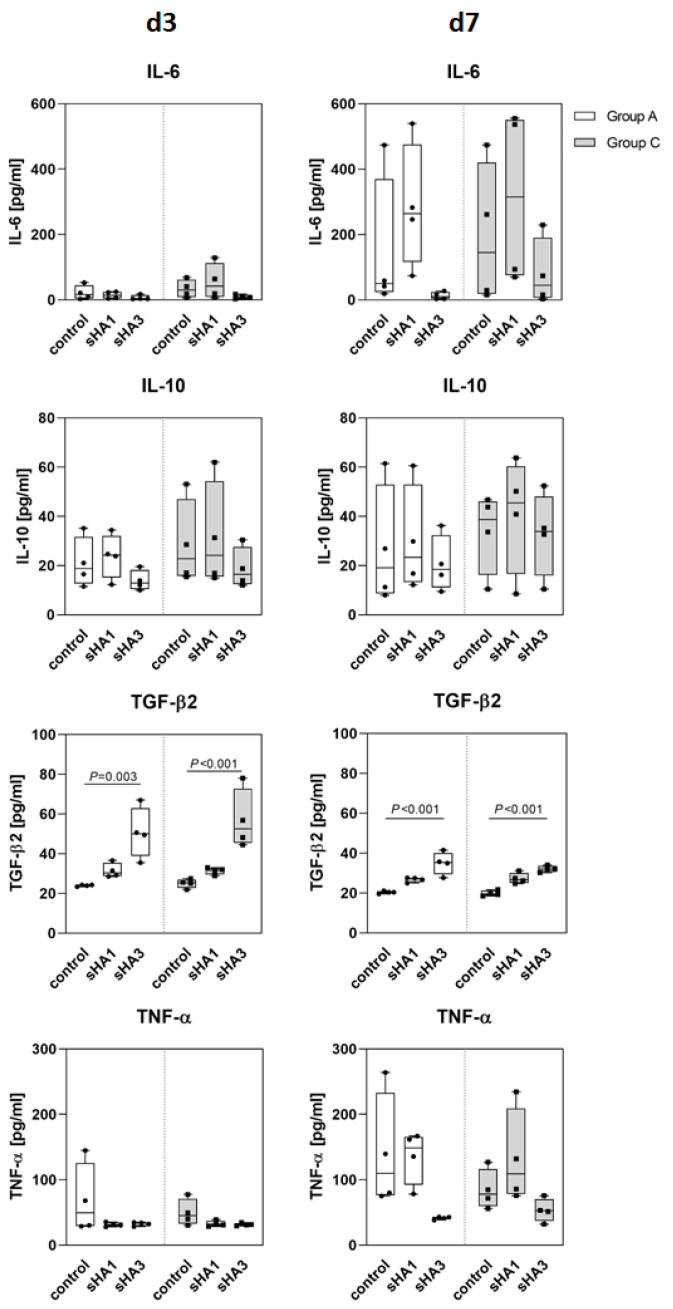
Impact of sHA1 and sHA3 on cytokine concentrations in PBMC of arthritis (group A) and Charcot patients (group C). PBMC were treated with M-CSF/RANKL and sHA derivatives. Cell culture supernatant was collected on day 3 and 7 for cytokine quantification by ELISA (n = 4). Data are given as median with interquartile range and min/max values, statistical analyses were performed using ANOVA and Tukey post-hoc test.

Results of ELISA analysis of PBMC cell culture supernatants at days 3 and 7 with and without sHA treatment for IL-6, IL-10, TGF-β2 and TNF-α are visualized in Figure 6. The basal concentrations of the cytokines did not vary significantly between groups. Noteworthy, the group C controls secreted slightly more IL-6 and IL-10 on day 3 than group A. IL-6 and IL-10 secretion of the control and sHA1-treated cells increased from day 3 to day 7 in both patient groups. PBMC treated with sHA3 released considerably less IL-6 and IL-10. TGF-β2 did not vary in the untreated controls of group A and group C. Both sHA-derivatives increased the TGF-β2 secretion significantly in both patient groups at days 3 and 7. In particular, sHA3 treated PBMC released significantly more TGF-β2 on day 7. TNF-α concentration was higher in the untreated control of group A at both time points. Taken together, sHA1 increased the release of IL-6, IL-10, TGF-β2 and TNF-α in both patient groups, especially after treatment for 7 days. sHA3 treatment diminished the secretion of IL-6, IL-10 and TNF-α in both patient groups, but increased TGF-β2 significantly.

## 3. Discussion

Charcot neuroarthropathy (CN) is a devastating condition leading to gross deformity and/or instability at the foot and ankle with lasting functional disability and a high risk of amputation [2,3]. CN is a multifactorial disease with complicated diabetes being the most prevalent cause. The underlying mechanisms, however, are not yet completely understood [5,9]. The current, patient-based study was performed to further elucidate the alterations in the manifestation of the disease in bone cells and explore possible treatment strategies.

Blood-derived monocytes from arthritis and CN patients undergoing reconstructive foot and ankle surgery were cultured either with or without RANKL, a cytokine that induces the differentiation into osteoclasts. RANKL increased TRAP activity and calcium phosphate resorption in both groups. These markers of osteoclastogenesis were significantly increased in CN patients. Additionally, the expression of osteoclast-specific genes was induced.

PBMC of CN patients that were treated with M-CSF only, a cytokine essential for monocyte survival, showed stronger initial resorption activity than cells from arthritis patients. Although this difference was not statistically significant, the enhanced values for TRAP and resorption activity in group C indicate that PBMC derived from CN patients are pre-activated and are probably in an advanced osteoclast differentiation state. Induced by RANKL, osteoclasts of CN patients revealed a significantly higher resorption activity compared to arthritis patients. The expression of the calcitonin receptor gene *CALCR* was increased in CN patients. When sHA derivatives were added to osteoclasts, both resorption and *CALCR* expression were reduced. Physiologically, occupation of the calcitonin receptor by calcitonin, a signal peptide regulating calcium homeostasis, results in inhibited bone resorption by osteoclasts.

*ACP5* and *CTSK* gene expression is activated by RANKL–RANK interaction via downstream signal transduction pathways [17], explaining the similar expression pattern we found for both genes. Since recombinant RANKL was constantly added to cell culture, expression of *ACP5* and *CTSK* was less affected by sHA treatment. Cathepsin K is a protease secreted by osteoclasts to degrade the organic components of bone matrix, namely collagen type I, suggesting a correlation between *CTSK* expression and calcium phosphate resorption. Pennypacker et al. (2014) described decreased osteoclast activation and diminished calcium phosphate resorption after cathepsin K inhibition [18,19]. sGAG are known to interact with collagen. By preventing cathepsin K from cleaving the collagen fibers, sGAG inhibit matrix degradation by osteoclasts [20]. This explains the significantly decreased calcium phosphate resorption seen in our study. Since *CTSK* expression was increased by sHA treatment, the results suggest alternative mechanisms or targets of sHA requiring further studies to investigate.

Hyperglycemia in diabetics leads to formation of advanced glycation end products. Once bound to their receptors, those activate the synthesis of M-CSF, which initiates osteoclast formation, and pro-inflammatory cytokines such as TNF-α [21]. Consistent with this, we found elevated concentrations of IL-6, IL-10 and TNF-α secreted by osteoclasts derived from CN patients. After treatment with sHA the secretion of these pro-inflammatory cytokines decreased suggesting anti-inflammatory effects of sHA that were recently reported for chronic wounds [22] but not yet for bone-related diseases or injuries.

IL-6 is a pleiotropic cytokine that can act both pro- and anti-inflammatory and regulates other cytokines such as the pro-inflammatory TNF-α. In experiments with neutralizing antibodies against IL-6, TNF-α was also blocked. Moreover, in ovariectomized mice, TNF-α-mediated bone loss was inhibited by treatment with an IL-6 antibody [23]. In another ex vivo study, the number of osteoclasts expressing IL-6 and TNF-α was elevated in bone specimens from diabetes patients suffering from CN [24]. This corresponds to the resorption behavior of osteoclasts from CN patients observed in the present study. IL-6 concentrations correlated with the amount of secreted TNF-α and with calcium phosphate resorption and all three parameters decreased after sHA3 treatment. On the other hand, secretion of the anti-inflammatory mediator TGF-β2 increased after sHA3 administration indicating an additional direct anti-inflammatory effect.

TGF-β2 secretion was elevated in both patient groups after sHA treatment. As sHA3 has been shown to promote the interaction of TGF-β with its receptor to recruit precursor cells and support wound healing [25], the enhanced TGF-β2 secretion observed in our study may also indicate a favorable influence on bone regeneration.

In the present study, sHA1 induced cytokine secretion while sHA3 attenuated cytokine release. For inflammatory conditions in diabetes with CN, these findings may be useful for improving pharmacological intervention.

TNAP provides inorganic phosphate for hydroxyapatite generation and thus bone mineralization [26]. Osteoblasts from both patient groups showed similar TNAP activity confirming the findings by Starup-Linde that TNAP was not decreased in diabetes mellitus despite changes in bone quality [27]. sHA1 did not affect TNAP activity which contrasts with previously published results on osteoblasts derived from bone marrow stromal cells of healthy donors [13]. In our study, sHA3 treatment resulted in reduced TNAP activity in osteoblasts of both patient groups. As described previously, the pro-osteogenic effect of sHA derivatives strongly depends on the differentiation/maturation status of osteoblasts. Osteoblast precursor cells and early osteoblasts respond to sHA with substantially higher TNAP activity and calcium phosphate accumulation than mature osteoblasts or osteosarcoma cell lines [12]. In the present study, osteoblasts isolated from older, chronically diseased, multi-morbid individuals were used, which may explain the less pronounced impact of sHA.

To further estimate the influence of sHA on osteogenesis, we investigated the expression of osteogenic marker genes with and without sHA treatment. *RUNX2* expression was significantly decreased in CN. Because a correlation between low *RUNX2* expression and hypoxic conditions has been shown [28,29] and because hypoxia has also been reported in diabetes mellitus [30], it can be concluded that the reduced *RUNX2* expression is related to the hypoxic conditions in diabetic bone.

The expression of the osteocalcin encoding gene *BGLAP* showed a similar pattern in both patient groups. Since the *BGLAP* gene product undergoes posttranslational modifications to obtain carboxylated osteocalcin, gene expression provides only limited information about the actual concentrations of mineralization-active osteocalcin [31,32]. sHA treatment had an impact on *ALPL* expression indicating pro-osteogenic effects of sHA3. These positive effects on osteoblasts and mineralization may be caused by sHA-triggered changes in ECM assembly of osteoblasts [13] and by the sHA-mediated scavenging of the Wnt antagonists Dkk1 and sclerostin [33]. The role of these factors in bone metabolism in diabetic CN has to be elucidated in further studies.

Because of their negative charges, GAG interact with cytokines and growth factors that are presented to cells in order to enhance their function [34]. Alternatively, the interaction of GAG with mediators could prevent their receptor binding [35]. In both cases, GAG orchestrate the cellular response to these growth factors and cytokines by promoting or inhibiting the access to their receptors. In various bone-related pathologies such as osteogenesis imperfecta, rheumatoid arthritis and diabetes mellitus the physiological ECM composition is altered [36,37,38] affecting both growth factor presentation and neutralization of mediators or mediator gradient formation in the surrounding tissue. Increasing knowledge on how the ECM composition altered by these diseases may help to develop treatment strategies by applying artificial ECM components.

Our study is not without limitations. First, the patient groups are heterogeneous with both CN and end-stage osteoarthritis representing a broad spectrum of pathological changes. With the number of patients available, it is impossible to analyze further subgroups (such as posttraumatic vs. idiopathic arthritis) nor to correct for all possible confounders. On the other hand, to the best of our knowledge, this is the first analysis of osteogenic and osteoclastic activity in a sizeable number of patients on a biochemical/cellular and gene expression level. Second, we do not have a control group with healthy bone since sampling of intact bone from healthy subjects would not be allowed due to ethical reasons. Still, we excluded patients with relevant systemic diseases from the arthritis group to have a control group to CN patients with otherwise physiological bone metabolism, which was confirmed by analysis of the blood samples.

## 4. Materials and Methods

### 4.1. Patients and Sampling

Human bone fragments and blood samples were obtained from patients undergoing reconstructive foot and ankle surgery at our institution after informed consent. The study was approved by the institutional review board (approval no. EK 530122016, 4 January 2017). Samples were obtained from two patient cohorts: Group A (Arthritis) consisted of patients with degenerative or posttraumatic arthritis. Patients suffering simultaneously from arthritis and systemic diseases such as diabetes mellitus, hyperuricemia, immunodeficiency or chronic inflammatory diseases were excluded. Group C (Charcot) consisted of patients with Charcot neuroarthropathy (CN) due to diabetes mellitus. Patients with acute infections were excluded from the study. Patient’s characteristics of both groups are summarized in Table 1.

Patient’s blood was drawn for monocyte isolation followed by induction of osteoclastogenesis. Bone fragments obtained through debridement during reconstructive surgery were stored in 155 nM NaCl until outgrowth of osteoblasts.

### 4.2. Cell Culture

The calcium phosphate matrix used for the resorption studies was prepared as previously described [39]. Briefly, SaOS-2 cells were cultured in McCoy’s 5A medium (Biochrom, Holliston, MA, USA) with 15% heat-inactivated fetal bovine serum (Biowest, Riverside, MO, USA), 20 µg streptomycin/mL and 20 U penicillin/mL on 24 well plates until confluence. To induce osteogenic differentiation, the medium was changed to α-MEM containing 10% heat-inactivated fetal bovine serum supplemented with 2 mM glutamine (Sigma, Taufkirchen, Germany), 20 µg streptomycin/mL (Gibco, Waltham, MA, USA), 20 U penicillin/mL (Gibco), 10 mM β-glycerophosphate and 300 µM ascorbic acid (both Sigma). Medium was changed trice per week. After 25 days, cells were detached using 20 mM NH_4_OH.

PBMC were isolated from EDTA blood by incubation in ACK lysis buffer (155 mM NH_4_Cl; 10 mM KHCO_3_; 110 mM Na_2_EDTA, all Sigma) for 15 min followed by centrifugation at 14,000 rpm for 5 min. Cells were resuspended in α-MEM (Sigma) with 20% heat-inactivated fetal bovine serum, 2 mM glutamine, 20 µg streptomycin/mL, 20 U penicillin/mL and with either M-CSF only or M-CSF and RANKL (both Peprotech) to induce osteoclastogenesis. A quantity of 2 × 10^5^ cells was seeded on SaOS-2-derived matrix for TRAP activity measurement and resorption assay or on TCPS for gene expression studies.

Bone fragments were crushed up using a bone crusher. The fragments were washed twice in sterile PBS with 20 µg streptomycin/mL and 20 U penicillin/mL and kept in a cell culture flask completely covered by α-MEM with 20% heat-inactivated fetal bovine serum, 2 mM glutamine, 20 µg streptomycin/mL and 20 U penicillin/mL for osteoblast outgrowth. Medium was changed once a week. At confluence, cells were detached by trypsin/EDTA and either cryopreserved or seeded for analysis.

### 4.3. Osteoclast Characterization

Tartrate-resistant acid phosphatase (TRAP) activity was determined in cell lysates after 14 days of culture. PBMC were lysed in lysis buffer containing 10 mM Tris (pH 7.5), 150 mM NaCl, 2 mM EDTA, 1% Triton X-100, aprotinin and PMSF (all Sigma). Quantities of 10 µL of cell lysate and 100 µL of TRAP reaction buffer (100 mM sodium acetate; 50 mM tartrate, both Sigma) containing p-nitrophenyl phosphate as a substrate were incubated at 37 °C for 1 h. By adding 100 mM NaOH the reaction was stopped. Then the optical density was assessed photometrically at 405 nm. TRAP activity was calculated from a linear calibration curve prepared with p-nitrophenol.

Osteoclast calcium phosphate resorption was determined as described elsewhere [39]. Briefly, 14 days after seeding on Saos-2 matrix the cells were washed with PBS and detached using 20 mM NH_4_OH. For von Kossa staining, the calcium phosphate matrix was incubated with 5% silver nitrate for 1 h and was then stained with 1% pyrogallol. Silver replaces calcium in the SaOS-2 matrix and appears black, while areas of resorption remain colorless. After scanning with transmitting light (600 dpi), image analysis was performed using Image J software. Binary images were generated to quantify the fraction of black and white areas in the wells.

### 4.4. Osteoblast Characterization

For tissue non-specific alkaline phosphatase (TNAP) activity determination osteoblasts were lysed after 14 days of cell culture using lysis buffer (10 mM Tris, 150 mM NaCl, 2 mM EDTA, 1% Triton X-100, protease inhibitors (1% aprotinin, 1% PMSF)). A quantity of 10 µL of lysate was incubated with 100 µL of reaction buffer (100 mM diethanolamine (Sigma); 0.1% Triton X-100) with p-nitrophenyl phosphate for 30 min at 37 °C. The reaction was stopped by adding 100 mM NaOH. The optical density was measured at 405 nm. Using a calibration curve prepared with p-nitrophenol, TNAP activity was calculated.

### 4.5. sGAG Synthesis

**Figure 7:**
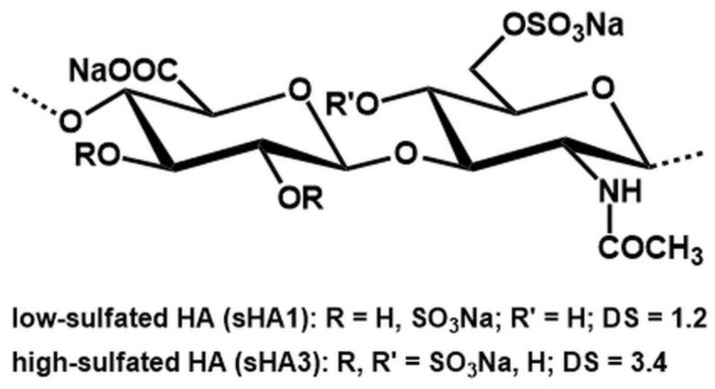
Structural characteristic of sulfated GAG derivatives.

High molecular weight hyaluronan (HA, isolated from *Streptococcus*, Mw = 1,200,000 g mol^−1^ kDa) was purchased from Aqua Biochem (Dessau, Germany). As educt for sulfation we used thermally degraded HA derivatives synthesized according to standard protocols [40]. The sHA derivatives with low and high degree of sulfation (DS; average number of sulfate groups per repeating disaccharide unit; sHA1: 1.2; sHA3: 3.4) were synthesized as described previously [40]. For the preparation of low-sulfated HA (sHA1) the ratio of OH:SO_3_ was 1:3.5 and the reaction time was 20 min; for the synthesis of high-sulfated HA (sHA3) we used the ratio of OH:SO_3_ = 1:15 with a reaction time of 60 min. SO_3_/pyridine was used as the sulfating reagent. Derivatives were characterized by elemental analysis and nuclear magnetic resonance (NMR). Determination of molecular weight was performed by gel permeation chromatography (GPC) with a triple detection system consisting of laser light scattering (LLS) detector, a refraction (RI-) and UV-detector. Absolute values of number-averaged (M_n_; sHA1: 12,704 g mol^−1^; sHA3: 54,376 g mol^−1^) and weight-averaged (M_w_; sHA1: 19,106 g mol^−1^; sHA3: 75,610 g mol^−1^) molecular weights were determined using the LLS detection system. Calculation of polydispersity (PD = M_w_/M_n_; sHA1: 1.96; sHA3: 1.58) was performed on the basis of M_n_ and M_w_ values obtained from RI detection. The chemical structures of respective sHA derivatives are shown in Figure 7. For cell experiments, 200 µg sHA/mL cell culture medium was added 2 h after cell seeding and with every medium change.

### 4.6. RNA Isolation, Reverse Transcription and Real-Time qPCR

RNA was isolated from cells after 14 days of culture using the peqGOLD MicroSpin Total RNA Kit (Peqlab, Erlangen, Germany). Reverse transcription with the High Capacity cDNA Reverse Transcription Kit (Applied Biosystems/Thermo Fisher Scientific, Waltham, MA, USA) was performed to synthesize cDNA, which was then applied for quantitative real-time PCR using the TaqMan Fast Advanced Master Mix (Applied Biosciences). The following TaqMan Gene Expression Assays (Applied Biosystems/Thermo Fisher Scientific, Waltham, MA, USA) were tested: *ACP5* (Tartrate-resistant acid phosphatase (TRAP)); *CTSK* (Cathepsin K); *CALCR* (Calcitonin receptor); *ALPL* (Tissue non-specific alkaline phosphatase (TNAP)); *BGLAP* (Osteocalcin); *RUNX2* (Runt-related transcription factor 2). Relative gene expression is given as ΔΔC_T_ relative to the house-keeping gene *GAPDH* [41].

### 4.7. Determination of Cytokine Release

Supernatants of PBMC culture were collected at every medium change and stored at −20 °C until further use. After thawing, the samples were centrifuged at 13,000 rpm and 4 °C for 30 min. Then Quantikine ELISA (R&D Systems, Wiesbaden, Germany) was performed for human IL-6, human IL-10, human TGF-β2 and human TNF-α according to manufacturer’s instructions.

### 4.8. Statistical Analysis

All cell culture experiments were performed in triplicates, real-time PCR and ELISAs were conducted in duplicates. If not stated otherwise, data are presented as boxplots showing median, interquartile range, min and max values. Graphs and statistics were prepared using GraphPad Prism 9.0 software. If not stated otherwise, all statistical analyses were calculated using 2-way analysis of variance (ANOVA) with Tukey post hoc test. *p* values of <0.05 were considered statistically significant. Exact *p* values are depicted within the figures.

## 5. Conclusions

In summary, we demonstrated increased osteoclast activity in complicated diabetes mellitus with CN through simultaneously elevated expression rates of resorption-related genes. Following sHA administration, both the resorption rate and the expression of osteoclast-specific genes decreased. The expression of osteogenic genes changed only marginally after sHA treatment suggesting a less pronounced impact of sHA derivatives on osteoblasts. However, cytokine release shifted towards a more anti-inflammatory state indicating positive effects of high sulfated hyaluronan on bone cells in CN. These results reveal further insights on the pathomechanisms of CN and potential treatment with ECM components. Further studies are warranted to elucidate the mechanisms of signal transduction to reveal how sHA affects intracellular processes and inflammatory events in bone and immunoregulatory cells.

## Figures and Tables

**Table 1:** Characteristics of included patients.

	Group A (Arthritis)	Group C (Charcot)
Number	26	17
Age (years)	56.1 ± 2.7	62.5 ± 2.2
Sex (women to men ratio)	14/12	2/15
C-reactive protein (mg/L)	4.1 ± 0.98	8.45 ± 1.97
Serum glucose (mmol/L)	5.73 ± 0.49	9.54 ± 1.29
HbA1c (%)	n.d.	6.7 ± 0.57

Values (except number and sex) shown as mean ± SEM. n.d.: not determined.

## Data Availability

The data presented in this study are available on request from the corresponding author. The data are not publicly available due to ethical reasons. Data sharing is not in accordance with consent provided by the participants.
